# λ-Carrageenan Oligosaccharides of Distinct Anti-Heparanase and Anticoagulant Activities Inhibit MDA-MB-231 Breast Cancer Cell Migration

**DOI:** 10.3390/md17030140

**Published:** 2019-02-27

**Authors:** Hugo Groult, Rémi Cousin, Caroline Chot-Plassot, Maheva Maura, Nicolas Bridiau, Jean-Marie Piot, Thierry Maugard, Ingrid Fruitier-Arnaudin

**Affiliations:** Equipe BCBS (Biotechnologies et Chimie des Bioressources pour la Santé), Université de La Rochelle, UMR CNRS 7266 LIENSs, 17000 La Rochelle, France; hugo.groult@univ-lr.fr (H.G.); remi.cousin1@univ-lr.fr (R.C.); caroline.chotplassot@etudiant.univ-lr.fr (C.C.-P.); maheva.maura1@univ-lr.fr (M.M.); nicolas.bridiau@univ-lr.fr (N.B.); jean-marie.piot@univ-lr.fr (J.-M.P.); thierry.maugard@univ-lr.fr (T.M.)

**Keywords:** λ-carrageenan, heparanase, anticoagulant, depolymerisation, cell migration

## Abstract

In tumor development, the degradation of heparan sulfate (HS) by heparanase (HPSE) is associated with cell-surface and extracellular matrix remodeling as well as the release of HS-bound signaling molecules, allowing cancer cell migration, invasion and angiogenesis. Because of their structural similarity with HS, sulfated polysaccharides are considered a promising source of molecules to control these activities. In this study, we used a depolymerisation method for producing λ-carrageenan oligosaccharides (λ-CO), with progressive desulfation over time. These were then used to investigate the influence of polymeric chain length and degree of sulfation (DS) on their anti-HPSE activity. The effects of these two features on λ-CO anticoagulant properties were also investigated to eliminate a potential limitation on the use of a candidate λ-CO as a chemotherapeutic agent. HPSE inhibition was mainly related to the DS of λ-CO, however this correlation was not complete. On the other hand, both chain length and DS modulated λ-CO activity for factor Xa and thrombin IIa inhibition, two enzymes that are involved in the coagulation cascade, and different mechanisms of inhibition were observed. A λ-carrageenan oligosaccharide of 5.9 KDa was identified as a suitable anticancer candidate because it displayed one of the lowest anticoagulant properties among the λ-CO produced, while showing a remarkable inhibitory effect on MDA-MB-231 breast cancer cell migration.

## 1. Introduction

In order to identify an alternative to conventional cancer chemotherapy, which is based on the inhibition of mitosis inmalignant cells, medical research has become interested in the specific biological attributes of the tumor microenvironment [1,2]. One of the distinctive features of this microenvironment is the overexpression of numerous hydrolytic enzymes, including an endo-β-d-glucuronidase called Heparanase (HPSE) [3]. HPSE is the only enzyme able to hydrolyse the heparan sulfate (HS) chains of proteoglycans at specific glycosidic bonds that are components of the extracellular matrix and cell-surfaces [4]. This activity governs matrix l integrity and basement membrane degradation, allowing cancer cell migration and invasion. It concurrently releases sequestered HS-binding growth factors, cytokines or enzymes, leading to inflammatory and angiogenic signalling activation [5,6]. The identification of drugs targeting HPSE that could be used as promising anticancer therapeutics has therefore been the subject of many studies [7].

Sulfated polysaccharides are widely appreciated as a potent class of inhibitors because they are structurally related to HS, a natural substrate of HPSE [8]. However, their high molecular weight (MW), complex structure and broad range of bioactivities, which could lead to unforeseen events, have limited their clinical development for this precise application. For instance, heparin (UFH), a close analogue of HS, is considered a gold standard for HPSE inhibition, however its well-known anticoagulant activity limits its use in oncology due to the risk of internal bleeding [9,10]. Consequently, efforts have been made to screen oligosaccharide derivatives suitable for in vivo use and with a potentiated anti-HPSE specificity. The synthesis of oligosaccharides derivatives is essentially based on depolymerisation processes as well as chemical modifications to vary the degree of sulfation (DS) and acetylation or to open carbohydrate rings using the glycol-split method [9,11]. Four compounds are currently being tested in clinical trials: two are derived from heparin (Roneparstat^®^ and Necuparanib^®^), one is a heterogeneous mixture of sulfomannan oligosaccharides (Muparfostat^®^) and the last is a synthetic tetrasaccharide conjugated to a steroid moiety (PG 545) [12]. However, their mechanism of action at the molecular level is not yet clearly understood [13]. Although the recent report showing for the first time the crystal structure of HPSE definitely constitutes a major breakthrough in the field that will lead to molecular modelling simulations [14], the structural heterogeneity of the candidates together with the challenging chemical carbohydrate synthesis remain obstacles to a detailed understanding of the specific units that mediate enzyme inhibition [15]. For instance, the role of glycol-split sugars used in several candidates is still under debate [16,17], while recent works have suggested a complex unusual mode of inhibition that varies with ligand concentration [18]. In addition, the bioactivities of oligosaccharide derivatives are clearly not restricted to the anti-HPSE activity and their anticancer effects are due to their interactions with other macromolecules, most notably growth factors [19,20]. Finally, in the case of heparin-based inhibitors, the difficulty of producing native heparin at a high yield constitutes a further complication, and the fact that they are of animal origin means that production has an environmental cost [21,22]. Thus, it is still necessary to screen natural polysaccharide derivatives and determine different production conditions in order to improve our understanding of the relationship between the carbohydrate architecture and the anti-HPSE function [23].

**Figure 1:**
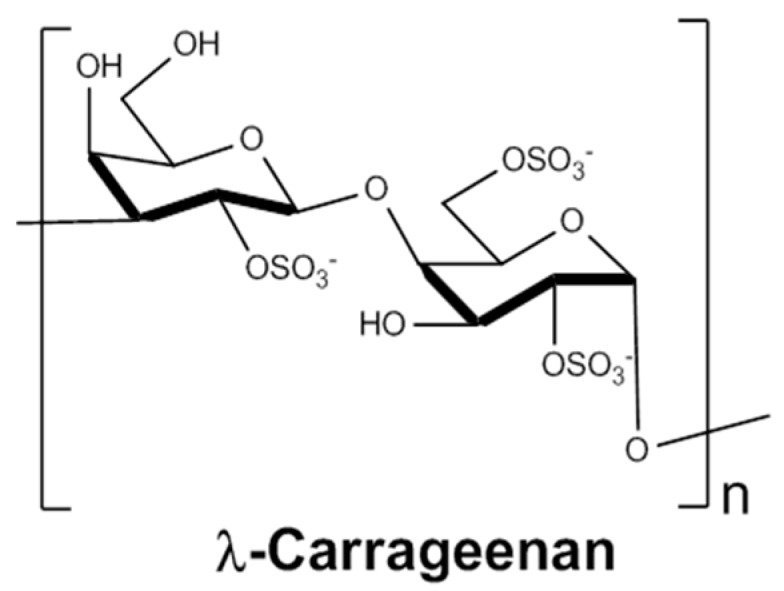
λ-carrageenan structure.

Carrageenans are a family of high MW sulfated galactans that are extracted from red seaweed in an environmentally-friendly way and are already extensively used in the food industry for their rheological properties [24]. They are characterised by long homogeneous linear chains of repeated disaccharide units consisting of a 1,3-linked β-d-galactopyranose (G unit) alternating with a 1,4-linked α-d-galactopyranose (D unit), differently sulfated depending on the species [25]. The G2S-D2S,6S disaccharide unit, bearing three sulfate groups, forms λ-carrageenans, which are known as the most sulfated plant-based polysaccharides with an ester sulfate content of about 35% in weight (Figure 1). Like other polysaccharides, carrageenans have many pharmacological properties, including their anticoagulant, antiviral, antioxidant or anticancer activities; these are summarized in the excellent review by Pangestuti et al. [26]. Again, this diversity limits their use to specific pharmacological applications in the clinic due to potentially serious adverse effects [27]. As a matter of fact, there is currently some debate over a pro-inflammatory and possible toxicity of the carrageenans used as food additives [28]. Fortunately, methods for modulating their bioactivities have been applied, with the main one involving various depolymerisation strategies [29]. In particular, three groups have reported anticancer activity of low MW λ-carrageenan derivatives [30,31,32]. These effects are mainly explained by a stimulation of the immune response [33,34,35], a direct cytotoxic effect [36,37] or an interaction with growth factors or associated receptors [31,38,39]. Initially hypothesized in some studies [40,41], an anti-HPSE activity has also been investigated recently [31,42,43]. However, most studies assessing the structure/activity of λ-carrageenan oligosaccharides have focused on the effect of polymer MW, however the exact role of each sulfate group in the anticancer effect, especially in HPSE inhibition, remains to be clarified. Moreover, the influence of the depolymerisation process on other bioactivities of λ-carrageenans that can limit their use as anticancer agents has barely been studied in parallel [41]. For instance, the anticoagulant properties of carrageenans, especially of the λ type, have been widely reviewed and could be one of these limitations [44].

In this work, we developed a scaling-up method for the depolymerisation of λ-carrageenan under two temperature conditions, with a partial desulfation according to the reaction time. The anti-HPSE and anticoagulant properties were assessed to determine the influence of λ-carrageenan polymeric chain length and the role of the sulfate groups. We identified a potential anticancer candidate that was assessed in vitro for the inhibition of MDA-MB-231 breast cancer cell migration.

## 2. Results and Discussion

### 2.1. Depolymerisation of λ-Carrageenan

**Figure 2:**
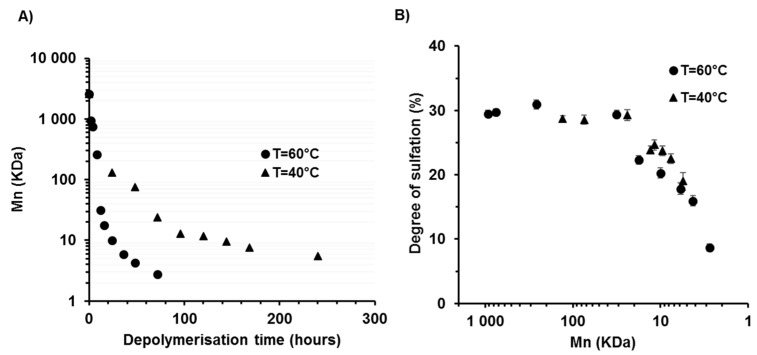
(**A**) Effect of the H_2_O_2_-assisted radical depolymerisation method on λ-carrageenan number average molecular weight (Mn) at 60 °C and 40 °C. (**B**) Degree of sulfation of λ-CO produced at 40 °C and 60 °C as a function of the Mn.

**Figure A1:**
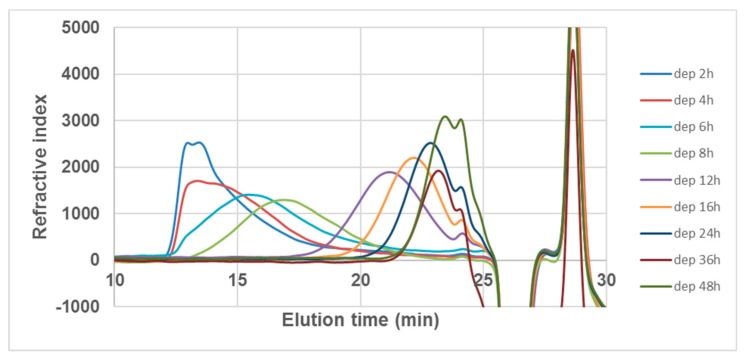
SEC-HPLC analysis with refractive detector of λ-CO produced along time. The SEC (size exclusion chromatography) separation was performed on a TSK-GEL G5000PW column in series with a TSK-GEL G4000PW at a flow rate of 0.5 mL/min using 0.1 M sodium nitrate (NaNO3) as the eluent.

**Figure A2:**
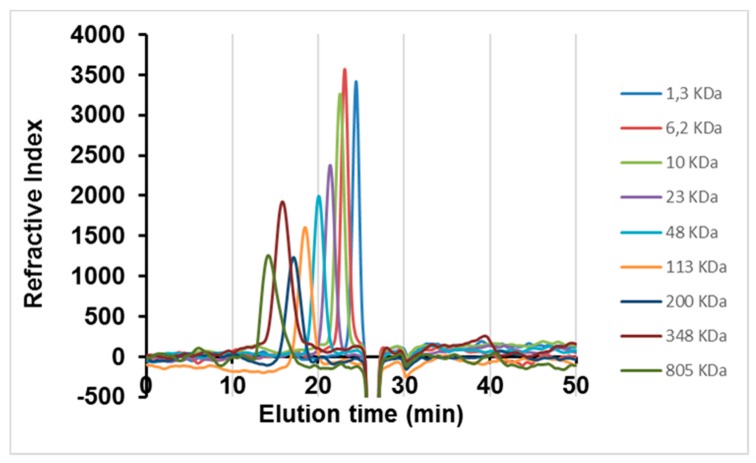
SEC-HPLC analysis with a refractive detector of pullulan standards of different molecular weights to construct the calibration curve. The SEC separation was performed on a TSK-GEL G5000PW column in series with a TSK-GEL G4000PW at a flow rate of 0.5 mL/min using 0.1 M sodium nitrate (NaNO3) as the eluent.

λ-carrageenans were depolymerised using a radical hydrolysis method with H_2_O_2_ at 40 and 60 °C. The H_2_O_2_/λ-carrageenan (*w*/*w*) ratio was set at 1.5, a ratio previously described as being suitable for obtaining low MW carrageenans [43]. The Mn of λ-CO produced over time was measured by SEC (size exclusion chromatography)-HPLC analysis (Table 1 and Appendix A, Figure A1). A calibration curve of pullulans was selected because, to the best of our knowledge, no carrageenan oligosaccharide standards are commercially available (Appendix A, Figure A2). Native λ-carrageenans are characterized by a high Mn of about 2586 kDa, corresponding to a DP of 4463, based on a reference weight of 579 Da per disaccharide unit. Depolymerisation was effective in both cases: there was an initial sudden fall in Mn within the first hours followed by a more progressive decrease, as shown by the logarithm scale used on the y-axis of Figure 2A. As expected, the kinetics of chain length reduction was faster at the highest temperature. Indeed, the Mn was reduced to 4.3 kDa after 48 h of reaction at 60 °C, which corresponds to a mean of 7 disaccharides units, while 240 h at 40 °C were needed to reach a similar level of depolymerisation (5.5 KDa). These results confirm previous work by our group showing that radical hydrolysis is an advantageous method for λ-carrageenan depolymerisation (Poupard, Groult, et al., 2017) and complements other techniques proposed such as acidic hydrolysis or the use of microwave protocols.

The effect of depolymerisation on the DS of λ-CO was then assessed (Table 1). Surprisingly, most sulfates were removed when the Mn was less than 20 kDa once the initial rapid kinetics of depolymerisation had slowed down. Thus, for an Mn ≥ 20 kDa, the effects of λ-CO Mn on various biological activities can be investigated separately from the influence of the sulfate groups. For an Mn below 20 kDa, a significant desulfation was observed. Indeed, native λ-carrageenans are characterized by a DS of ~30%, which is reduced to 8.7% for species with the lowest Mn, corresponding to a ~70% loss. This corresponds to a mean of 0.87 sulfate groups per disaccharide unit compared to the three sulfate groups bound to the native polymer. Overall, the DS was related to the Mn of the derivatives whatever the temperature condition although, in some cases, there was slightly less sulfate removal for derivatives prepared at 40 °C compared to those prepared at 60 °C (Figure 2B). It was concluded that the preservation of the DS was difficult to control by changes in the temperature condition with this depolymerisation method. Nevertheless, the slight differences will be used in subsequent structure/activity studies. This is of interest because the high DS of λ-carrageenans is often suggested to account for their better anticancer activity compared to the other types of carrageenans, however this aspect remains to be thoroughly investigated.

Thus, most previously published structure-activity relationship studies have focused on the influence of the length of oligosaccharide derivatives, while the outcome of the sulfation pattern has barely been addressed [30,31,32]. Navarro et al. previously reported that the 2-O sulfate position of the G unit is more resistant to hydrolysis [45]. Thus, we may assume that the desulfation observed may be distinguished by specific sulfate substitutions.

### 2.2. Effects of the Degree of Depolymerisation and Sulfation on the Anti-Heparanase Activity of λ-CO

**Figure 3:**
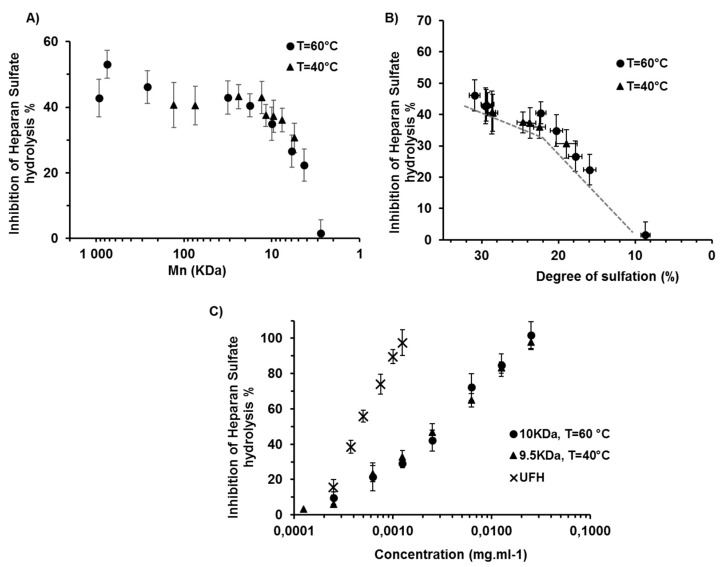
(**A**) Anti-HPSE (heparanase) activity of λ-CO produced at 40 °C and 60 °C as a function of their Mn for the inhibition of heparan sulfate hydrolysis by heparanase at 1.25 × 10^−3^ mg·mL^−1^. (**B**) Correlation between the DS of λ-CO produced at 40 °C and 60 °C and their inhibitory activity against heparan sulfate hydrolysis by heparanase. (**C**) IC_50_ of 10 kDa (produced at 60 °C, after 24 h) and 9.5 kDa λ-CO (produced at 40 °C, after 144 h) compared to native UFH (heparin); (HPSE = 100 ng·mL^−1^ and HS = 0.5 μg·mL^−1^).

We then studied the impact of polymer chain length and sulfate level on the anti-HPSE activity of λ-carrageenan derivatives. HPSE cleaves the β-glycosidic bonds of HS chains between a glucuronic acid (GlcA) and a glucosamine (GlcN) through a general acid catalysis mechanism which involves two glutamate residues. Two patches of basic amino acids at either side of the catalytic site, known as the heparin binding domains HBD-1 and -2, coordinate the interaction with HS sites of a specific sulfation pattern [4]. The inhibition of a recombinant HPSE by each oligosaccharide from a labelled-HS hydrolysis was monitored using a FRET (fluorescence resonance energy transfer)-based assay at a fixed concentration of 1.25 × 10^−3^ mg·mL^−1^ (Figure 3A). The anti-HPSE activity was maintained initially before it was to be reduced at ~20 kDa, which corresponds to the value at which desulfation starts. This indicated that the inhibitory activity was not correlated with the Mn of the derivatives, at least for those greater than 20 kDa. In fact, a rare lengthy structural pattern cannot be excluded as an explanation for the reduced activity of λ-CO below this Mn, although it is more likely to be the result of the decrease in the DS. Indeed, when the anti-HPSE activity was plotted against the DS, there was a clear correlation (Figure 3B). Interestingly, this activity seemed to be more drastically impaired when the DS was less than 20%, which corresponds to a mean loss of one sulfate group per disaccharide unit. In case of discriminated desulfation, this could indicate that the first preferentially removed sulfate substitution is not essential for enzyme inhibition. In line with this result, previous work on heparin has shown that the concurrent presence of sulfate groups at the O-2 position of IdoA and at the O-6 position of GlcN was not mandatory for an effective inhibition of HPSE [11].

We then compared the half maximal inhibitory concentrations (IC_50_) of two of our derivatives of about 10 kDa (24 h at 60 °C and 144 h at 40 °C) to that of native heparin, a gold standard for heparanase inhibition (Figure 3C). λ-CO had a moderate ability to inhibit HPSE with an IC_50_ about six times higher than that of native heparin, 3.1 mg·L^−1^ and 3.0 mg·L^−1^ versus 0.47 mg·L^−1^, respectively. Although they were less potent HPSE inhibitors than heparin, they may represent a promising alternative given heparin’s disadvantages, which include a costly and low-yield production, strong possibility of contamination and the fact that it is of animal origin, with the environmental issue that entails.

### 2.3. Effects of the Degree of Depolymerisation and Sulfation on the Anticoagulant Activity of λ-CO

**Figure 4:**
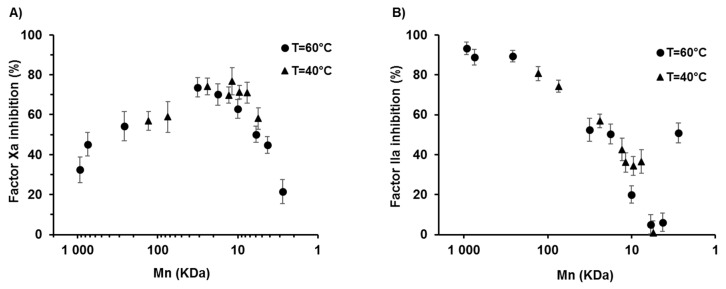
(**A**) Anti-Factor Xa activity of λ-CO produced at 40 °C and 60 °C as a function of their Mn, at 0.025 mg·mL^−1^ and (**B**) Anti-Factor IIa activity of λ-CO produced at 40 °C and 60 °C as a function of their Mn, at 0.125 mg·mL^−1^ (AT III = 0.625 μg·μL^−1^ and factor Xa or IIa = 11.25 nK at·mL^−1^).

**Figure A3:**
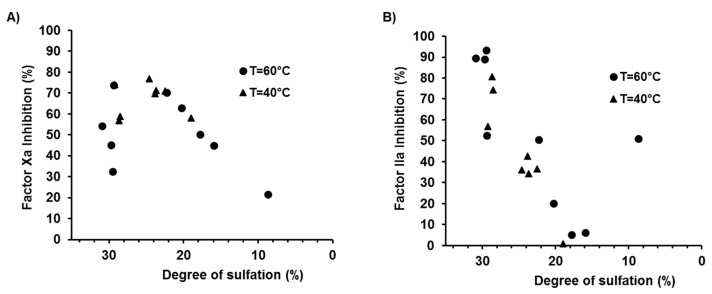
(**A**) Correlation between the DS of λ-CO produced at 40 °C and 60 °C and their inhibitory activity against Factor Xa and (**B**) Correlation between the DS of λ-CO produced at 40 °C and 60 °C and their inhibitory activity against Factor IIa.

A λ-carrageenans have been shown to have a higher anticoagulant activity compared to other members of the carrageenan family, although this activity is much weaker than that of heparin, a reference in this field [24,46]. Their anticoagulant activity has been attributed mainly to the inhibition of thrombin IIa and Factor Xa mediated by anti-thrombin III (AT-III) and/or heparin cofactor II [44]. The interactions between carrageenans and these plasma cofactors are complex and the functions of various parameters have been extensively discussed, including saccharide composition, MW, charge density, DS and sulfate position [47,48,49]. The anticoagulant properties of the carrageenan derivatives prepared in this study were thus studied through their ability to inhibit Factors Xa and IIa via AT-III activation. These key factors intervene at the end of the coagulation cascade to activate fibrin formation, which will then polymerize to form blood clots. To discuss the results, a mechanism similar to that of heparin was hypthesized. This consists of an AT-III conformational activation through binding with a specific pentasaccharide sequence present in the heparin chain, and the resulting complex inhibits Factors Xa and IIa. Regarding Factor IIa, an additional steric hindrance effect caused by the other sugars of a sufficiently long heparin chain is involved [50]. The anti-Xa activity of each oligosaccharide was measured at 0.025 mg·mL^−1^ by following the initial velocity for converting a chromogenic substrate compared to a control (vehicle). An increase in anti-Xa activity associated with the initial reduction in chain length was first observed for the derivatives (Figure 4A). This could be due to a higher steric freedom gained from the start of the depolymerisation of the very long native polymer that allows a better accessibility to the probable binding sequence of carrageenan to AT-III. Then, from 20 kDa, a decrease in anti-Xa activity was observed that was clearly related to the desulfation of λ-CO (Appendix A, Figure A3). For example, the 17.8 kDa and 7.5 kDa derivatives with an equivalent DS of ~22.5% had the same anti-Xa activity of about 70%. This result suggests that sugars with key sulfate substitutions are included in the potential binding sequence of λ-carrageenans to AT-III. Moreover, it appeared that in this case, the three sulfate positions had to be present because the correlation showed that anti-Xa activity was linearly impaired from the start of desulfation (Appendix A, Figure A3). In the literature, the role of the sulfate positions about the anticoagulant properties of λ-carrageenan is still under debate. Two previous studies have shown that sulfation at the C2 position of the D unit were beneficial to the anticoagulant activity [51,52].

Regarding factor IIa, overall the activities were lower than those of factor Xa and monitoring was performed at 0.125 mg·mL^−1^. As shown in Figure 4B, anti-IIa activity was first maintained before it rapidly decreased from 50 kDa, well before the start of desulfation. This result could be explained by the fact that depolymerisation decreases the additional steric effect needed for factor IIa inhibition, which is due to the long polymeric chains. Similarly, the thorough study by Melo et al. stressed that a MW higher than 45 kDa is required for the interaction between galactan oligosaccharides and factor IIa during the time of binding to AT-III [49]. Thus, although desulfation contributed to a decrease in anti-IIa activity by modifying the sequence by which λ-carrageenans bind to AT-III, the role of the polymeric chain length/Mn appeared to be more significant (Appendix A, Figure A3). Finally and somewhat surprinsingly, anti-IIa activity was recovered for the smallest oligosaccharide that was produced at 60 °C (2.77 kDa; 31.5% inhibition) and was almost completely desulfated. λ-CO did not inhibit factor Xa or factor IIa in the absence of AT III (negative control) except for derivatives of less than 6 kDa. This suggests that an oligosaccharide chemically defined by very low sulfate substitutions on the galactopyranose units was obtained and that it had a direct anti-thrombin activity. Other studies have shown that other oligosaccharides were likely to interact directly with the proteins of the coagulation cascades without potentiation of AT-III [53,54]. Taken together, the results showed that, although anti-IIa activity was rapidly abolished for λ-CO of less than 50 kDa, the Mn range of 20–500 kDa induced anti-Xa properties and should not be used for the development of a λ-carrageenan-based anticancer candidate to avoid any adverse effects like internal bleeding.

### 2.4. Effect of λ-CO Candidates on the Migration of MDA-MB-231 Breast Cancer Cells In Vitro

**Figure 5:**
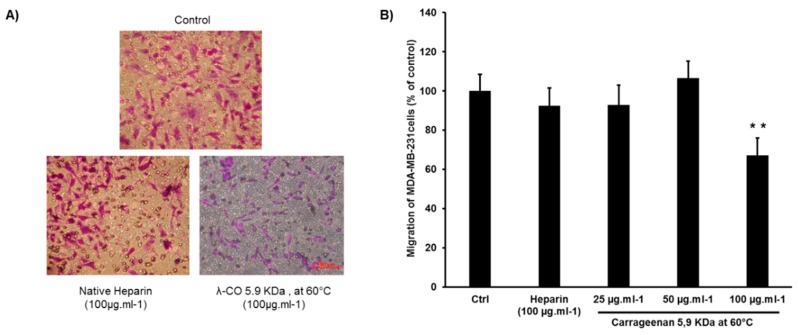
(**A**) Images showing the effects and (**B**) the inhibition activity at three doses 25, 50 and 100 µg·mL^−1^ of the λ-CO candidate 5.9 KDa prepared at 60 °C, compared with native heparin (at 100 µg·mL^−1^) on the migration ability of human breast MDA-MB-231 cells. The migration ability was assessed in a transwell assay after 24 h as described in Materials and Methods. The data are representative as the mean (±SEM of the errors mean) from three independent experiments, with at least four replicates. ** *p* < 0.01, test Anova-Two way Bonneferonni with mean ± SD of the three independent experiments.

**Figure A4:**
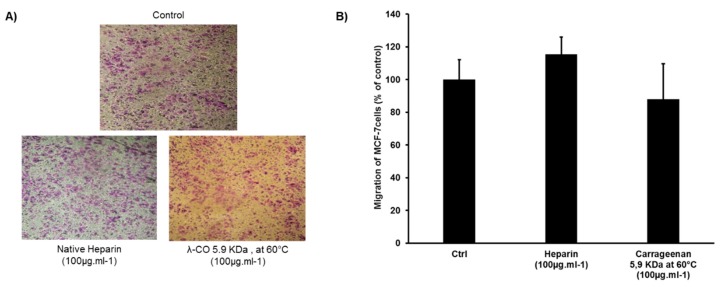
(**A**) Images showing the effects and (**B**) the inhibition activity of the λ-CO candidate 5.9 KDa prepared at 60 °C compared with native heparin (at 100 µg·mL^−1^) on the migration ability of human breast MCF-7 cells. The migration ability was assessed in a transwell assay after 24 h, as described in Materials and Methods. For Heparin, the data are representative as the mean (±SEM of the errors mean) from three independent experiments, with at least four replicates. For λ-CO, the data are representative as the mean ± SD of one experiment, with at least four replicates.

Since HPSE activity has been shown to be involved in the metastatic potential of cancer cells, the effect of a λ-CO derivative (5.9 KDa produced at 60 °C) on HPSE-associated migration were compared to native heparin using a transwell assay [55,56]. The highly motile MDA-231 breast cancer cells with high-level expression of HPSE were selected for this experiment [42,57]. As shown in Figure 5A, large number of control cells (treated with vehicle) passed through the pores towards the lower chambers in response to a 10% FBS (Fetal Bovine Serum) solution that was used as a chemoattractant. Treatment with native heparin led to a moderate 7.6% inhibition rate in accordance with previously published results [58,59,60]. Treatment with the λ-CO candidate at 100 μg·mL^−1^ significantly reduced the migration of MDA-MB-231 cells. The inhibition rate (32.8%) was higher than with heparin, while heparin had the best IC50 against HPSE activity (Figure 5B). The experiment was also repeated on the more quiescent MCF-7 breast cancer cell line (Appendix A, Figure A4) [61]. In this case, heparin slightly promoted the MCF-7 cells migration. Though, here, the λ-CO candidate again displayed an inhibitory activity of 12% on MCF-7 cell migration, however this was non-significant. As anticipated, this revealed that in addition to HPSE, the λ-CO candidate probably interacts with other molecules involved in the motility of cancer cells. It is known that migration is regulated by a complex interplay between varied glycosaminoglycans (e.g., syndecan-4 at the cell surface), protein expressions and degradative enzymes, in which heparin may have a different impact [62].

## 3. Materials and Methods

All reagents, unless otherwise specified, were purchased from Sigma Aldrich (Saint Louis, MO, USA). Native λ-carrageenans were purchased from FMC Biopolymer (Villefranche-Sur-Saône, France).

### 3.1. Depolymerisation of λ-Carrageenan for the Production of Oligosaccharides (λ-CO)

Native λ-carrageenans were dissolved in 200 mL of Milli-Q water at a concentration of 5 mg·mL^−1^. The solution was rapidly heated to 40–50 °C to completely dissolve the polysaccharide and then purged under argon. Then, 30% hydrogen peroxide (H_2_O_2_) (5 mL) was added and the reaction mixture was immediately sealed and placed in an incubator at 40 °C or 60 °C under 200 rpm stirring. Aliquots were taken at different time points and were dry frozen prior to analysis.

### 3.2. Structural and Quantitative Analysis of λ-CO by Size Exclusion Chromatography (SEC)

Structural and quantitative analysis of λ-CO by size exclusion chromatography (SEC) were performed using a LC/MS-ES system from Agilent (Santa Clara, CA, USA) (1100 LC/MSD Trap VL mass spectrometer) with two columns, TSK-GEL G5000PW and TSK-GEL G4000PW (30 cm × 7.5 mm), mounted in series. The columns were maintained at 30 °C and the products were eluted with 0.1 M Sodium nitrate (NaNO_3_) at a flow rate of 0.5 mL·min^−1^. The products were detected and quantified by differential refractometry using HP Chemstation software (Agilent, Santa Clara, CA, USA). Pullulans of different molecular weights ranging from 1.3 to 805 kDa purchased from Polymer Standards Service GmbH (Mainz, Germany) were used as calibrants for the standard curve and to determine the size of the carrageenan derivatives. The number-average molecular weights (Mn), weight-average molecular weights (Mw) and polydispersity index (PI) were calculated according to a previously published method [63] using the following equations:

(1) $$M_{n}=\frac{(\sum N_{i}\times M_{i})}{\sum N_{i}}$$

(2) $$M_{w}=\frac{(\sum N_{i}\times {M_{i}}^{2})}{(\sum N_{i}\times M_{i})}$$

(3) $$\mathrm{PI}=\frac{M_{w}}{M_{n}}$$

with *N_i_* representing the number of moles of polymer species and *M_i_* the MW of the polymer species. The degree of polymerization (DP) was calculated as follows:

(4) $$DP=\frac{M_{n}}{M_{0}}$$

with *M*_0_ representing the G2S-D2S,6S disaccharide unit MW set at 579 Da.

### 3.3. Quantification of the Sulfation Degree of λ-CO

The DS was monitored using (7-aminophenothiazin-3-ylidene)-dimethylazanium chloride (Azure A), which binds the sulfated groups on the sugar backbone to form a colored complex [64]. In a 96-well plate, 20 μL of three dilutions (0.03, 0.04 and 0.05 mg·mL^−1^) of λ-CO samples were added to 200 µL of a 10 mg·L^−1^ aqueous Azure A solution. Absorbance was measured at 640 nm after 10 min of incubation. DSs were calculated from a calibration curve constructed using absorbance values obtained from a serial dilution (0–0.03 mg·mL^−1^) of a dextran sulfate standard with a known sulfur content of 17%.

### 3.4. Anti-Heparanase Activity of λ-CO

The inhibition of HPSE activity was assessed using the heparanase assay toolbox (Cisbio Assay, Codolet, France) and heparanase purchased from R&D systems (HPSE-1 human recombinant heparanase). Briefly, upon excitation at 337 nm, a HS substrate labeled with both biotin and Eu3+ cryptate can produce a fluorescent emission at 665 nm by fluorescence resonance energy transfer (FRET) to streptavidin-XL665 (SA-XL665), which is added during the detection step. During hydrolysis, HPSE cleaves the substrate, resulting in a loss of possible energy transfer and thus, a reduction in SA-XL665 emissions. The enzymatic reaction was performed in white 96-well half-area plates (Corning^®^ #3693) and was monitored using a spectrofluorometer (BMG Labtech FLUOstar Omega, Champigny-sur-Marne, France) with a high time resolved fluorescence (HTRF) module. First, 15 µL of λ-CO or heparin solutions in Milli-Q water were added into the wells followed by 15 µL of heparanase solution (HPSE-1, 400 ng·mL^−1^ in Tris-HCl at pH 7.5, 0.15 M NaCl and 0.1% CHAPS). After a 10-min pre-incubation at 37 °C, an enzyme reaction was initiated by adding 30 µL of a Biotin-HS-Eu(K) solution (1.0 ng·µL^−1^ in 0.2 M sodium acetate buffer, pH 4.5) and the plate was incubated at 37 °C for 15 min. At the end of the reaction, the detection step consisted of adding 30 µL of streptavidin-XL665 solution (SA-XL665, 10 ng·µL^−1^ in NaPO_4_ 0.1 M buffer, pH 7.4, 0.8 M KF, 0.1% BSA, 1 mg·mL^−1^ heparin). The fluorescence was measured after 5 min at λ_em1_ = 620 nm and λ_em2_ = 665 nm, after 60 µs of excitation at λex = 337 nm. The Delta F (%) was calculated using the following equation according to the manufacturer’s instructions:

(5) $$\mathrm{Delta}\;F\;(\%)=\frac{\left(F665/F620\right)\;sample-\left(F665/F620\right)\;blank}{\left(F665/F620\right)\;blank}\times 100$$

with F665 and F620 representing the fluorescence signals measured at 665 nm and 620 nm, respectively. The percentage of inhibition was calculated based on the Delta F(%) of the maximum heparanase activity measured in the absence of inhibitor. The HPSE activity of each *λ-CO* was measured at a final concentration of 1.25 × 10^−3^ mg·mL^−1^. For the IC_50_ calculations, a curve-fitting tool from SigmaPlot software (Systat Software Inc, San Jose, CA, USA) was applied using a sigmoidal, logistic three-parameter equation.

### 3.5. Anticoagulant Activity of λ-CO

For anti-Xa and anti-IIa activity assays, 25 μL of λ-CO solution in Milli-Q water were incubated with anti-thrombin III (25 μL, 0.625 μg·μL^−1^) at 37 °C in 96-well plates for 2 min. Then, factor Xa or factor IIa was added at a final concentration of 11.25 nKat·mL^−1^ (25 μL). After 2 min of incubation, 3.25 nM (25 μL) of factor Xa chromogenic substrate (CBS 31.39; CH2SO2-D205 Leu-Gly-Arg-pNA, AcOH) for the anti-Xa activity assay or 1.4 nM (25 μL) of factor IIa chromogenic substrate (CBS 61.50; EtM-SPro-Arg-pNA, AcOH) for the anti-IIa activity assay were added. Absorbance of the reaction mixture was read for 3 min at 405 nm every 8 s with an absorbance reader (FLUOstar Omega BMG Labtech, Champigny-sur-Marne, France). The initial velocity was determined as the slope of the linear segment of the kinetics curve and the % of inhibition was calculated based on the initial velocity of an inhibitor-free blank (Milli-Q water). Anti-Xa and anti-IIa activities of each λ-CO were measured at a final concentration of 0.025 mg·mL^−1^ and 0.125 mg·mL^−1^, respectively. Controls used to assess a direct inhibition of Factors Xa or IIa were performed with the same protocol, however the anti-thrombin III solution was replaced by a vehicle solution (Milli-Q water). Native heparin at the same concentrations has been used as a positive control for 100% inhibition to validate the assay.

### 3.6. MDA-MB-231 Cell Migration Assay

A total of 1 × 10^5^ MDA-MB-231 or MCF-7 breast tumor cells were seeded in serum-free DMEM medium in the upper chambers of a transwell plate (8-µm pore size; Stardest). After 24 h, the medium was replaced by fresh serum-free DMEM medium treated with the polysaccharides candidates at concentration between 25 and 100 µg·mL^−1^ or the vehicle (Milli-Q water); meanwhile, a complete DMEM medium with 10% FBS was added to the lower chambers. After a 24 h culture at 37 °C, the cells that had migrated to the lower chambers were fixed with cold ethanol and stained with 0.1% crystal violet. Then, the non-migrating cells were removed from the upper chambers by wiping the membrane with cotton swabs. The remaining cells were photographed and eluted with 10% acetic acid solution. The absorbance of the resulting dilution was measured at 600 nm with an absorbance reader (FLUOstar Omega BMG Labtech, Champigny-sur-Marne, France). The percentage of inhibition was calculated by the following equation:

(6) $$\mathrm{Inhibition}\;(\%)=(1-\frac{A_{sample}}{A_{blank}})\times 100$$

with *A_sample_* representing the absorbance of 10% acetic acid solutions obtained from the cells treated with the polysaccharides candidates and *A_blank_* representing the absorbance obtained from the cells treated with the vehicle.

## 4. Conclusions

We developed a radical hydrolysis method for an easy large-scale production of structurally varied low MW λ-carrageenan derivatives. According to the depolymerisation temperature and time, λ-CO with distinct Mn and DS can be obtained. This is relevant for structure/activity relationship studies since previous studies have only focused on the Mn of the derivatives without really discussing the role of the sulfate substitutions of λ-carrageenans. HPSE inhibition was assessed and it was mainly correlated with the DS of λ-CO without a clear correlation with the Mn. It also appeared that one of the three sulfates of the λ-carrageenan disaccharide unit may not be essential for HPSE inhibition. Further experiments are needed to assess not only the effect of the overall DS of species, however also the specific role of each sulfate substitution. The development of λ-carrageenan-based anticancer candidates could be limited by unexpected anticoagulant properties, which were therefore assessed. Unlike HPSE inhibition, interactions between λ-CO and coagulation factors Xa and IIa were correlated with both the Mn and the DS. Moreover, while most species inhibited factors Xa and IIa through their interaction with AT-III, the smallest desulfated λ-CO was identified as a direct thrombin inhibitor. It would be interesting to screen other bioactivities of λ-carrageenans that could be related to adverse effects in oncology, including their pro-inflammatory properties.

We identified a suitable anticancer drug candidate with an anti-heparanase activity, the 5.9 kDa λ-CO produced after 36 h at 60 °C. At 60 °C, its production was more rapid than at 40 °C and its size is small enough to be appropriated for in vivo applications. Although its DS is slightly below the threshold DS for effective HPSE inhibition, at the same time, its partial desulfation (17%) and size guarantee not to fall within specifications for optimum λ-CO anticoagulant properties. Moreover, in a preliminary anticancer biological assessment, it showed very promising activity against MDA-MB-231 cell migration. To complete this finding, the study should be extended to other targets involved in tumor development and that are likely to interact with λ-CO, in particular the HS-sequestered molecules, such as growth factors.

## Figures and Tables

**Table 1:** Physicochemical properties of λ-CO. Molecular weight (Mn and Mw), Degree of polymerisation (DP) and degree of sulfation (DS).

Condition	Time (h)	Mn (KDa)	Mw (KDa)	DP	I	DS% (*w*/*w*)
**Depolymerisation at 60 °C**	0	2585.9	2722.2	4463	1.1	~30
	2	931.3	1120.2	1607	1.2	29.5 ± 0.5
	4	752.5	987.2	1299	1.3	29.7 ± 0.5
	8	261.4	425.8	451	1.6	30.9 ± 0.7
	12	31.7	42.7	55	1.4	28.0 ± 0.6
	16	17.8	23.8	31	1.3	22.3 ± 0.7
	24	10.0	12.7	17	1.3	20.3 ± 0.8
	36	5.9	8.4	10	1.4	17.8 ± 0.9
	48	4.3	6.8	7	1.6	15.9 ± 0.8
	72	2.8	3.7	5	1.4	8.7 ± 0.6
**Depolymerisation at 40 °C**	0	2585.9	2722.2	4463	1.1	~30
	24	130.3	197.2	225	1.5	27.6 ± 0.5
	48	74.5	110.4	129	1.5	27.6 ± 0.7
	72	23.9	36.2	41	1.5	29.2 ± 0.8
	96	13.0	19.2	22	1.5	23.8 ± 0.6
	120	11.7	17.4	20	1.5	24.6 ± 0.8
	144	9.5	14.3	16	1.5	23.7 ± 0.8
	168	7.6	11.2	13	1.5	22.5 ± 0.8
	240	5.5	8.0	10	1.4	19.0 ± 1.4
